# Chronic Statin Administration May Attenuate Early Anthracycline Associated Declines in Left Ventricular Ejection Function

**DOI:** 10.1016/j.cjca.2014.11.020

**Published:** 2014-11-26

**Authors:** Runyawan Chotenimitkhun, Ralph D’Agostino, Julia A Lawrence, Craig A. Hamilton, Jennifer H. Jordan, Sujethra Vasu, Timothy L. Lash, Joseph Yeboah, David M. Herrington, W. Gregory Hundley

**Affiliations:** 1 Department of Internal Medicine (Cardiology Section), Wake Forest School of Medicine, Winston-Salem, North Carolina; 2 Department of Public Health Sciences, Wake Forest School of Medicine, Winston-Salem, North Carolina; 3 Department of Internal Medicine (Hematology and Oncology Section), Wake Forest School of Medicine, Winston-Salem, North Carolina; 4 Department of Biomedical Engineering, Wake Forest School of Medicine, Winston-Salem, North Carolina; 5 Department of Radiology, Wake Forest School of Medicine, Winston-Salem, North Carolina; 6 Department of Epidemiology, Rollins School of Public Health, Emory University, Atlanta, Georgia

**Keywords:** statin, heart failure, anthracycline

## Abstract

**Background:**

Recent studies show an association between statin therapy and a reduced risk of heart failure among breast cancer survivors. Our goal was to evaluate whether statin therapy for prevention of cardiovascular disease (CVD) would ameliorate declines in left ventricular ejection fraction (LVEF) often observed during anthracycline-based chemotherapy (Anth-bC).

**Methods:**

In 51 participants (33 women and 18 men; aged 48±2 years), we performed CV magnetic resonance (CMR) measurements of LVEF before and 6 months after initiation of Anth-bC for patients with breast cancer, leukemia, or lymphoma. Fourteen individuals received statin therapy, and 37 received no statin. MR image analysts were blinded to participant identifiers.

**Results:**

Those receiving statins were older and often had diabetes (DM), hypertension (HTN), and hyperlipidemia (HLD). For those receiving statins, LVEF was 56.6±1.4% at baseline and 54.1±1.3% 6 months after initiating anthracycline (*p*=0.15). For those not receiving a statin, LVEF was 57.5±1.4% at baseline and decreased to 52.4±1.2% over a similar 6 month interval (*p*=0.0003). In a multivariable model accounting for age, sex, DM, HTN, HLD, and cumulative amount of anthracycline received, LVEF remained unchanged in participants receiving a statin (+ 1.1±2.6%) versus a −6.5±1.5% decline among those not receiving a statin (p=0.03).

**Conclusion:**

In conclusion, these data highlight that individuals receiving statin therapy for prevention of CVD may experience less deterioration in LVEF upon early receipt of Anth-bC than individuals not receiving a statin. Further studies with large numbers of participants are warranted to determine if statins protect against LVEF decline in patients receiving Anth-bC.

## Introduction

Anthracycline-based chemotherapy (Anth-bC) is an important component of adjuvant chemotherapy for breast cancer and an essential element of curative combination chemotherapy for acute leukemia, Hodgkin’s disease, non-Hodgkin’s lymphoma, and many other solid tumors.^1,2^ The cytotoxic anti-tumor effects from Anth-bC are related to their interactions with the enzyme topoisomerase IIα, production of double strand DNA breaks, and the generation of intracellular cytotoxic free radicals.^3^ Unfortunately, in cardio-myocytes, these cytotoxic free radicals promote oxidative and nitrosative stress that, in combination with other anthracycline related effects (systemic inflammation and neuro-hormonal activation), promote left ventricular dysfunction, myocardial replacement fibrosis, congestive heart failure, and cardiovascular (CV) events.^4–14^ Strategies that could reduce Anth-bC mediated myocellular oxidative/nitrosative stress could diminish LV dysfunction and possibly improve overall cancer-related survival.

Several lines of evidence suggest that generic, inexpensive, oral 3-hydroxy-3-methylglutaryl-coenzyme A (HMG-CoA) reductase inhibitors (statins) may attenuate cardio-myocyte injury during and after receipt of Anth-bC.^15^ While this class of drugs is commonly used to treat hypercholesterolemia, they also reduce oxidative and nitrosative stress, inflammatory cytokines, and circulating neuro-hormones.^16,17^ In a recent observational study, women receiving statins for primary or secondary prevention of CV events who also received adjuvant chemotherapy for breast cancer experienced fewer heart failure (HF) related billing code events than women receiving similar breast cancer therapy without concomitant statin use.^18^

Based on the above considerations, we hypothesized that participants receiving anthracycline chemotherapy who were also taking statin therapy for primary or secondary prevention of CV events may experience smaller decreases in left ventricular ejection fraction (LVEF) when compared to individuals not taking statins. To test this hypothesis, we measured LVEF with cardiovascular magnetic resonance (CMR) before and 6 months after initiation of Anth-bC in 51 participants with breast cancer, leukemia, or lymphoma.

## Materials and Methods

### Study Population and Design

The study was approved by the Institutional Review Board of the Wake Forest University School of Medicine and all participants provided witnessed written informed consent. Between 2007 and 2010, we enrolled 51 consecutive participants who were recruited from the hematology and oncology outpatient and inpatient facilities of the Comprehensive Cancer Center at Wake Forest Health Sciences and scheduled to receive Anth-bC. Of the cohort enrolled, we separated participants into two groups: 14 individuals that were receiving statins for primary or secondary prevention of CV events, and 37 individuals who were not receiving a statin.^19,20^ Each participant was scheduled to receive a CMR measurement of LVEF on 2 occasions: before receipt of their Anth-bC and then 6 months after initiation of chemotherapy. All acquired images were transferred to workstations for determination of LVEF and mean mid-wall circumferential myocardial strain by personnel blinded to participant identifiers, study group, and the date or results of the other CMR examination (a blinded, unpaired read).

### CMR image acquisition analysis

Images were acquired with a 1.5-T Magnetom Avanto Scanner (Siemens, Munich, Germany) whole body imaging system using a phased-array cardiac surface coil according to previously published techniques.^21,22^ These sequences incorporated steady-state free-precession cine white blood imaging techniques in which a series of short axis slices were positioned across the LV apical four-chamber view beginning at the LV base and terminating at the LV apex. Imaging parameters included a 34 cm field of view, a 47.3 ms repetition time (TR), a 1.1 ms echo time (TE), an 80° flip angle (FA), an 8 mm thick slice with a 2 mm interslice gap, and a 192x109 matrix. The measurements of LVEF were performed according to previously published techniques.^23,24^

Tagged CMR images for calculation of myocardial strain were acquired in the middle LV short axis plane according to previously published methods using spatial modulation of magnetization (SPAMM).^25^ Imaging parameters included a 36 cm field of view, a 42 ms TR, a 3.8 ms TE, a 12° FA, an 8 mm thick slice, and a matrix size of 192x144. Mean, mid-wall LV circumferential strain (Ecc) was measured in the mid-ventricular short axis plane from the SPAMM grid tag deformations that occurred throughout the systolic frames according to previously published methods using harmonic phase (HARP) analyses (Diagnosoft, Raleigh, NC).^26^

### Statistical Analysis

Descriptive statistics were estimated for measures of interest including means and standard errors for continuous measures, and counts and percentages for categorical measures. For each continuous measure, a 2-sample t-test was performed to compare the statin users with non-statin users; for categorical measures, Fisher’s exact tests were performed to compare the two groups. Comparisons within the groups were made for changes in the LV function measures using paired t-tests. Comparisons between the groups (statin and non-statin users) were made using analysis of covariance (ANCOVA) models where the outcome was the change in LVEF after 6 months. Two models were created. In the first, the covariates included age, sex, height, weight, anthracycline dose, and an indicator variable for the number of CV co-morbidities present including high blood pressure, previous coronary artery disease, diabetes or tobacco use. The second model included the same demographic and chemotherapy variables, but rather than include the CV co-morbidities, considered the number of cardio-active medications received by participants including angiotensin-converting enzyme inhibitors, angiotensin receptor blockers, beta blockers, calcium antagonists and diuretics used to treat these co-morbidities. To determine if there was a dose response effect regarding the use of a statin, users of statins were grouped into high (40–80 mg daily) versus low (10–20 mg daily) dose groups and the ANCOVA model was re-fitted with the 3-level categorical dose variable included. All analyses were performed using SAS Version 9.2 and a p-value <0.05 was considered significant.

## Results

Demographic data from the 51 participants are displayed in Table 1. The age averaged 48±2 (range 19 to 71) years. The majority of participants were women (65%). Statin recipients were prescribed 40±5 mg (range 5 mg to 80 mg) of atorvastatin (n=5) or simvastatin (n=9). Those prescribed statins were older and more often exhibited diabetes (DM), hypertension (HTN), and hyperlipidemia (HLD) relative to those not receiving statins (Table 1), and they received more cardiovascular-related medications than non-statin users.

The indication for anthracycline chemotherapy did not differ respectively between statin users and non-users: with breast cancer (36% vs. 49%), lymphoma (36% vs. 35%), and leukemia (28% vs. 16%, p ≥ 0.59 for all). The anthracycline administered to participants for treatment of their malignancy included doxorubicin (n=38), daunorubicin (n=11), epirubicin (n=2), and idarubicin (n=1), according to established protocols;^27^ one participant received two of these agents, and the others received one of these agents. None of the participants received radiation therapy during the course of receipt of their anthracycline. The cumulative anthracycline dose in doxorubicin equivalent doses^28^ ranged from 30 to 450 mg/m^2^ and averaged 193±27 mg/m^2^ and 193±15 mg/m^2^ in statin and non-statin users, respectively (p=0.99).

**Figure 1:**
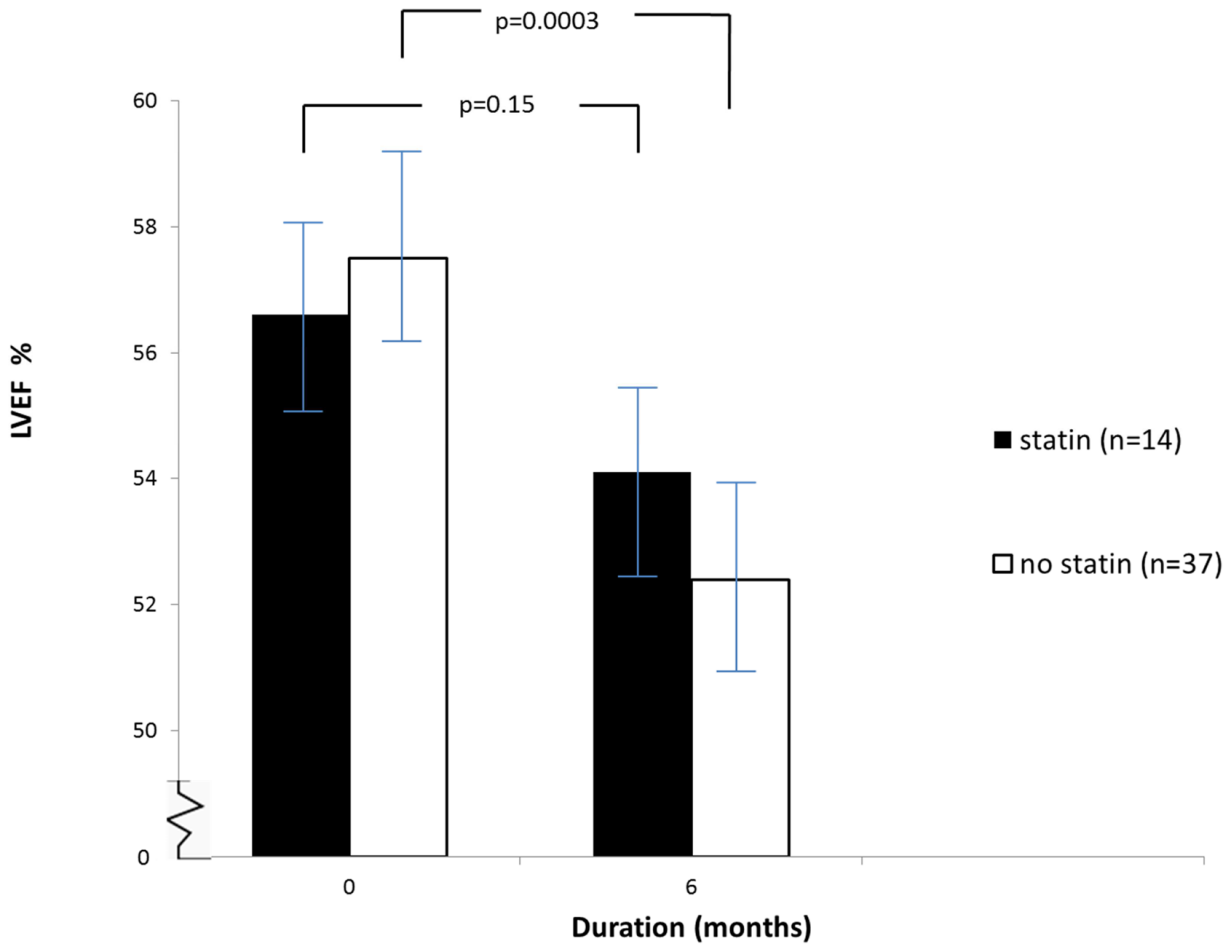
Measurement of left ventricular ejection fraction in patients receiving anthracyclines The mean ± the standard error of measures of left ventricular ejection fraction for the participants, (y-axis) and the time course (months) between study acquisitions (x-axis) are shown. Those prescribed statins (black bars) demonstrated no significant decrease in LVEF (p=0.14), whereas those not prescribed a statin (white bars) demonstrated a decrease in LVEF 6 months after receipt of anthracycline-based chemotherapy.

Before receipt of Anth-bC, without adjusting for differences in participant demographics, the baseline parameters of LV volumes were similar for statin and non-statin users (unadjusted; p=0.27 to 0.66 for all). The baseline LV mid-wall circumferential myocardial strain trended lower for statin users versus non-statin users (−16.9±0.8 versus −18.7±0.5, respectively, p=0.07). A somewhat similar trend in resting LVEF was also observed between the two groups (p=0.14). As shown in Figure 1, for the 14 individuals receiving statins, LVEF was 56.6±1.4% at baseline and 54.1±1.3% 6 months after initiating an anthracycline (p=0.15). For those not receiving a statin, LVEF was 57.5±1.4% at baseline and decreased to 52.4±1.2% over the same 6 month interval (p=0.0003).

In a multivariable model accounting for age, sex, DM, HTN, HLD, and the cumulative amount of anthracycline received, LVEF remained unchanged in participants receiving a statin (+ 1.1±2.6%) versus a −6.5±1.5% decline among those not receiving a statin (p=0.03). The baseline and 6-month measures of LV volume and myocardial strain after accounting for age, sex, height, weight, DM, HTN, smoking, hyperlipidemia, and doxorubicin equivalent dose are shown in Table 2. The data presented in Table 2 has also been adjusted for BMI. At the 6-month follow-up, LV mass decreased from baseline in both groups. In statin users, LV end diastolic volume index, LV end systolic volume index, and LV stroke volume index increased at the 6-month CMR exam relative to baseline. In non-statin users, LV end diastolic volume index remained similar, LV end systolic volume index increased, and LV stroke volume index decreased at 6 months relative to baseline.

**Figure 2:**
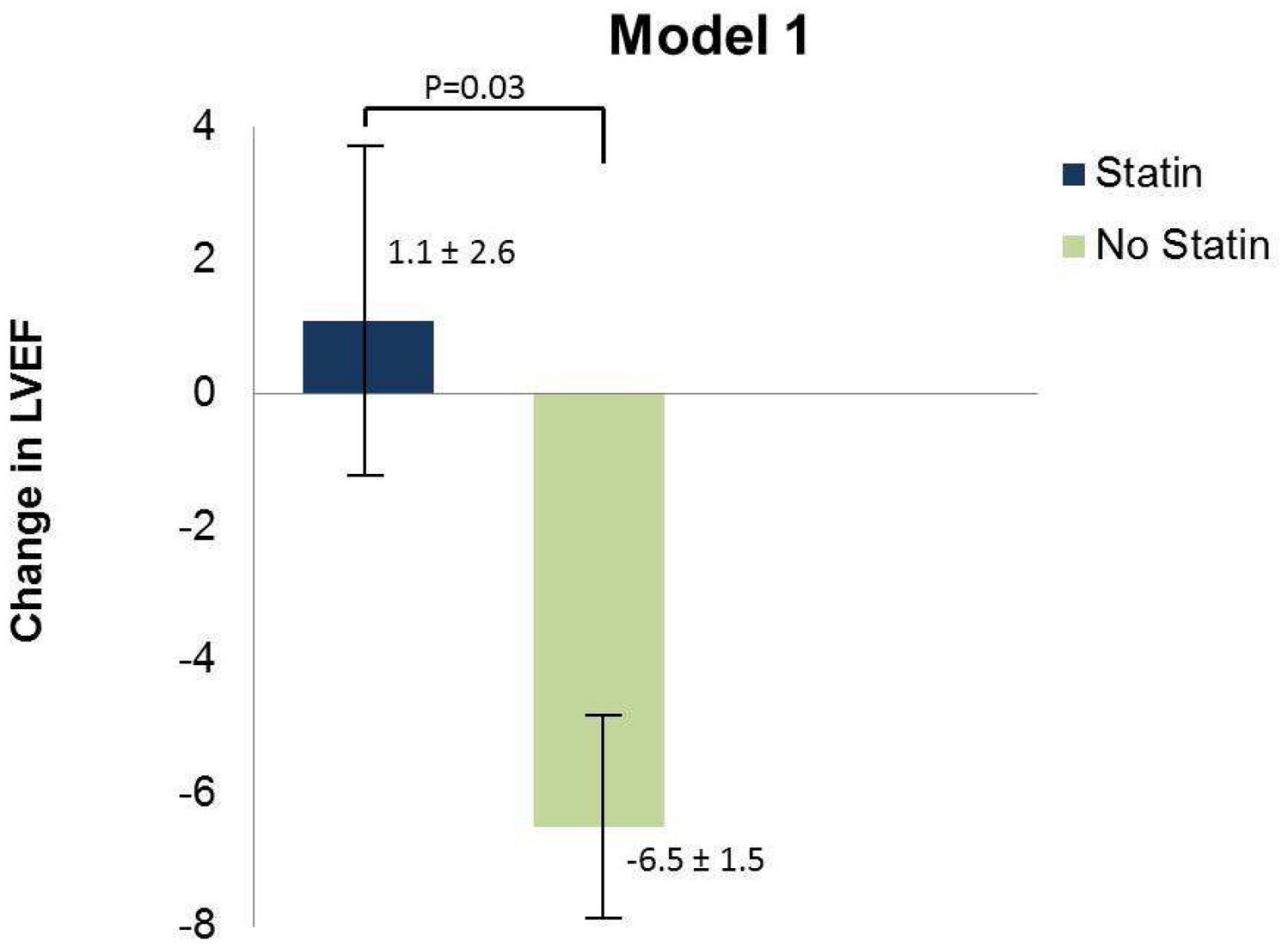

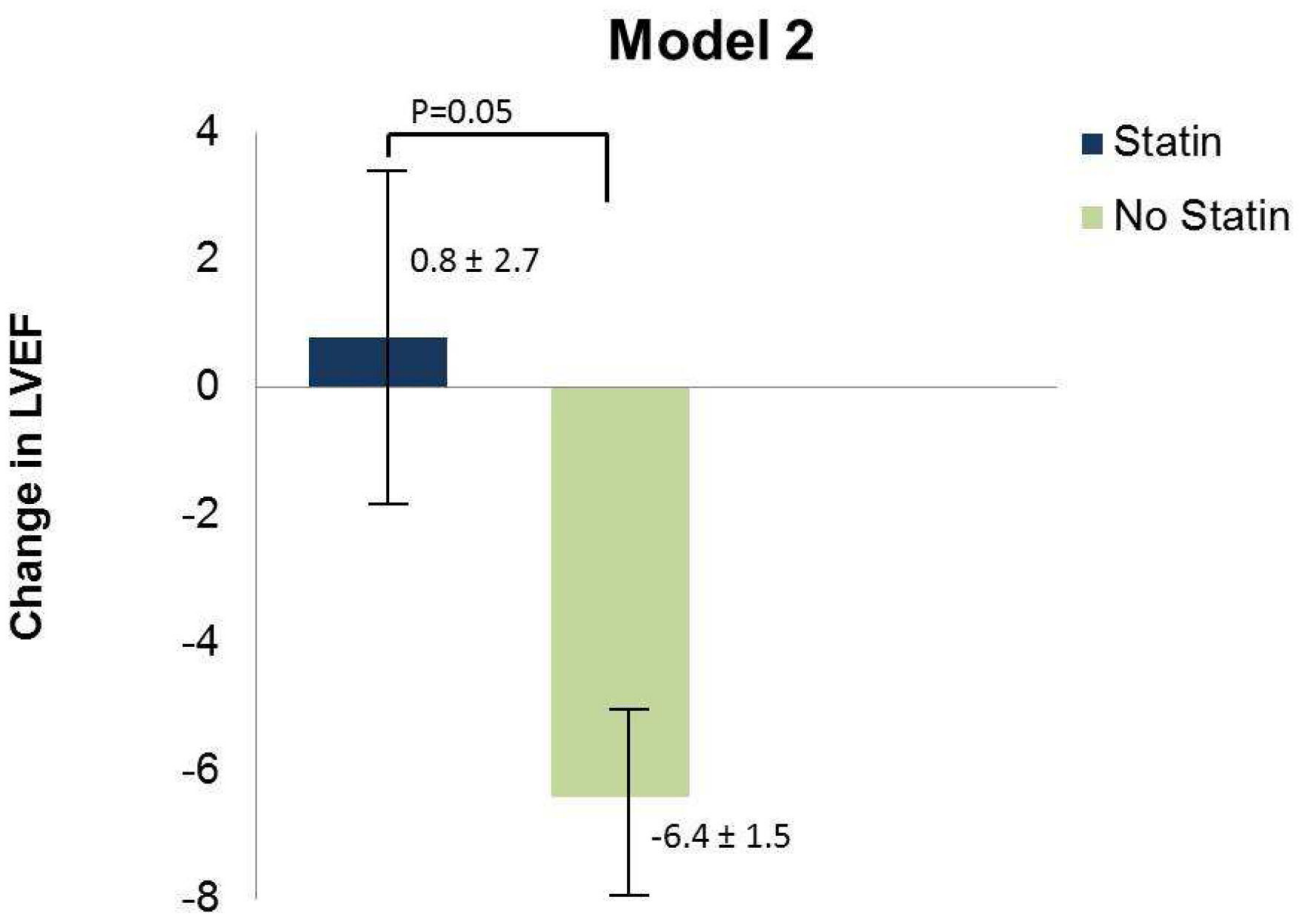
Change in LVEF in statin and non-statin users after accounting for potential confounding clinical variables Bar graphs demonstrate the mean ± standard error of change in LVEF from baseline to 6 months in statin and non-statin users. Model 1 (p=0.03) accounts for age, sex, BMI, accumulated dose of anthracycline, and the number of CV comorbidities (defined as diabetes, hypertension, smoking, coronary artery disease) that are present. Model 2 (p=0.05) accounts for age, sex, BMI, accumulated dose of anthracycline and potentially cardioactive medications such as beta blockers, ACE inhibitors/ARB, calcium channel blockers, and diuretics. As shown, after accounting for potential factors that influence LVEF other than the administration of anthracycline-based chemotherapy, those participants receiving statins experience less of a decline in LVEF relative to those not receiving a statin.

In the multivariable model accounting for age, sex, the comorbidities of DM, HTN, and HLD, tobacco use, height, weight and the cumulative doxorubicin equivalent dose, the LVEF increased slightly for statin users (1.1±2.6%) whereas there was a decline of −6.5±1.5% in LVEF for the non-statin users (p=0.03; Model 1, Figure 2). Similarly, after accounting for demographic variables, anthracycline doses, and cardio-active medications, the receipt of statins (LVEF increase of 0.8±2.7) remained associated with a preservation of LVEF relative to those not receiving statins (LVEF decrease of −6.4±1.5; p=0.05; Model 2, Figure 2).

In those receiving 40 to 80 mg/day of a statin, there was an increase in LVEF, whereas there was a 3.4±4% decrease in LVEF after low (10 to 20 mg/day) dose statin use, and a 9.2 ±3% decrease in LVEF in those not receiving a statin (p=0.02). When we tested these groups for a linear dose response, we found a highly significant effect (p=0.006). For mid-wall LV myocardial strain we observed decline of −2.9 ± 2.9 (with more negative values equating with improved strain) for the high dose statin users compared to a −0.7 ± 2.7 change for the low dose statin users and a 0.1 ± 2.6 increase for the non-statin users, suggesting a dose response relationship for this outcome as well. However, this difference did not reach statistical significance (p=0.33).

## Discussion

The results of this study indicate that patients prescribed statins for primary or secondary prevention of CAD that go on to receive Anth-bC treatment for their cancer experience a decline in LVEF early in the course of their treatment. However, after accounting for other factors that influence LVEF, participants receiving statins experienced smaller declines in LVEF, suggesting the possibility that statins may protect against precipitous declines in LVEF after receipt of Anth-bC (Figures 1 and 2). In addition, individuals receiving higher doses of statins (40 to 80 mg/day) may exhibit less deterioration in LVEF after receiving Anth-bC than those receiving smaller doses of or no statin therapy at all.^29–34^

As shown in Table 1, those prescribed a statin for primary or secondary CV event prevention (independent of their cancer or cancer treatment) were, as expected, generally older and exhibited more CV co-morbidities than those not receiving statins. The fact that LVEF (p=0.14) and LV mid-wall myocardial circumferential strain trended lower (p=0.07) before initiation of Anth-bC in those receiving statins is likely explained by a combination of the demographic (e.g., advanced age) and co-morbidities experienced by the statin recipients.

As shown in Figure 1, using the unadjusted data, LVEF declined to a greater extent in those not receiving versus receiving statin therapy. To account for the differences in the two groups, we performed ANCOVA using two models. The first model included demographic, chemotherapy treatment and CV co-morbidity co-variables. Since the receipt of the CV medications was highly correlated with the presence of CV morbidities, a second model that included demographics, chemotherapy and the medications used to treat the co-variables was constructed. The pre-and 6 month post-Anth-bC metrics of LV volumes, EF, and strain in first model indicate that LVEF declined in those not receiving a statin (Figure 2). Also shown in Figure 2 is the difference in LVEF deterioration observed between the statin and non-statin users persisting in the second model; this indicates that the receipt of a statin was associated with a smaller decline in LVEF early after receipt of Anth-bC, even after accounting for other potentially beneficial effects associated with other cardio-protective agents, such as ACE inhibitors or beta blockers. Systolic blood pressure and diastolic blood pressure were similar among the two groups throughout the study (p = 0.06 to 0.99 for all).

Interestingly, a high statin dose (40–80 mg/day) recipient experienced a small increase in LVEF relative to those receiving a low dose (10–20 mg/day) or no statin. This occurred even though those receiving high dose statin therapy also possessed a worse CV risk factor profile than those receiving low dose or no statin therapy. Potential mechanisms by which statins could preserve LVEF include their ability to reduce oxidative and nitrosative stress, inflammatory cytokines, and circulating neuro-hormones. Further potential mechanisms by which statins prevent declines in LVEF may include presentation of endothelial function in the coronary arterial microcirculation. The statins improved endothelial function and increased nitric oxide bioavailability on the coronary blood flow, resulting in increased myocardial tissue oxygen level. Statins could be another potential mechanism to prevent the decline of LV EF in patients who receive statins during Anth-bC.^35,36^ This finding is somewhat similar to decreases in myocardial apoptosis and myocyte death observed in rats receiving high versus low doses of alorvastatin in combination with doxorubicin therapy.^37^ Together with the data from this study, these observations raise the possibility that there may be a dose-dependent effect of the statins on anthracycline mediated CV injury.

Our study has the following limitations. First, the participant groups were somewhat dissimilar with respect to underlying demographic and comorbid conditions that could influence LVEF. To account for these differences, we implemented analysis of covariance (ANCOVA) models that incorporated variables known to influence LVEF. Although residual confounding could still be present in our study, the fact that a statistically significant difference was seen in this relatively small study suggests that the actual magnitude of the effect may be quite large. Second, while these observational results demonstrate an association between receipt of statin use and attenuation of LVEF declines after Anth-bC, one cannot infer causality. The data from this study, in combination with other recent publications indicating no increased incidence of breast cancer or its recurrence in women receiving statins, provide the background information necessary to initiate a randomized controlled clinical trial to determine if statins do, in fact, reduce the incidence of CV injury upon receipt of Anth-bC for breast cancer.^38–40^ Third, while the high accuracy and reproducibility of MRI measures allowed us to identify changes in LVEF in a relatively small sample size, the small number of participants exhibit limited biologic variability and thus we cannot evaluate the extent to which our results can be impacted by factors such as race or the presence of CV co-morbidities.

## Conclusions

In conclusion, the results of this study indicate that individuals treated with anthracycline-based chemotherapy for cancer who are also prescribed statin therapy for primary or secondary prevention of cardiac events at the time chemotherapy is initiated may experience a smaller decline in LVEF 6 months later when compared to those not receiving a statin. This apparent attenuation in anthracycline-related LVEF decline in statin users occurs even though they possess more risk factors for future cardiac events than non-statin users. These data suggest that further studies are warranted to determine if the prospective administration of statins attenuate LV dysfunction and CV events frequently associated with the administration of anthracycline-based chemotherapy.

## Figures and Tables

**Table 1:** Demographic data

Characteristics	Statin (n=14)	Non-Statin (n=37)	*p*-value
**Age, years**	62±2	43±2	<0.001
**Men:Women**	6:8	12:25	0.53
**Weight, lbs**	202±14	181±9	0.21
**Height, in**	68±1	67±1	0.84
**Body mass index, kg/m^2^**	30.7±1.6	27.7±1.1	0.14
**Co-morbid disease**			
Diabetes	7 (50%)	2 (5%)	<0.001
Hypertension	12 (86%)	10 (27%)	<0.001
Hyperlipidemia	14 (100%)	2 (5%)	<0.001
Smoking	9 (64%)	14 (38%)	0.12
Coronary artery disease	2 (14%)	1 (3%)	0.18
Myocardial infarction	1 (7%)	1 (3%)	0.48
**Cancer Type**			
Hematologic malignancy	9 (64%)	19 (51%)	0.53
Non-hematologic malignancy	5 (36%)	18 (49%)	
**Accumulative Dose of Anthracyclines and Derivative Agents, mg/m^2^**			
Doxorubicin	153±35	159±20	0.88
Daunorubicin	45±25	64±22	0.63
Epirubicin	36±36	9±8	0.30
**Other Chemotherapy Agents**			
Cyclophosphamide	9 (64%)	30 (81%)	0.27
Tamoxifen	0 (0%)	1 (3%)	1.0
Trastuzumab or Herceptin	2 (14%)	5 (14%)	1.0
**Other Medications**			
Beta blockers	7 (50%)	3 (8%)	0.003
ACE inhibitors	6 (43%)	4 (11%)	0.02
ARB	4 (29%)	0 (0%)	0.004
Calcium channel blockers	4 (29%)	1 (3%)	0.02
Diuretics	7 (50%)	1 (3%)	0.0002

Values expressed as n (%) and mean ± standard error.

Abbreviations: ACE, angiotensin converting enzyme; ARB, angiotensin II receptor blocker.

**Table 2:** Cardiac measurements adjusted for age, sex, body mass index, doxorubicin equivalent dose, and risk factors for cardiac events (diabetes, hypertension, smoking, and coronary artery disease).

Characteristics	Baseline	Follow-up	*p*-value
**Statin (n=14)**			
LV myocardial mass (gm)	127±8	112±8	0.16
LVEDV (mL)	109±9	118±8	0.33
LVESV (mL)	48±5	55±5	0.19
LV stroke volume (mL)	61±4	64±4	0.61
LVEDVi (mL/m^2^)	52±4	57±4	0.24
LVESVi (mL/m^2^)	23±2	27±2	0.12
LV stroke volume index (mL/m^2^)	29±2	31±2	0.51
Myocardial strain (mid-level:Eu)	−16±1	−15±1	0.33
**Non-Statin (n=37)**			
LV myocardial mass (gm)	119±5	113±5	0.16
LVEDV (mL)	122±5	122±5	0.93
LVESV (mL)	54±3	59±3	0.07
LV stroke volume (mL)	69±3	63±3	0.11
LVEDVi (mL/m^2^)	62±2	61±2	0.92
LVESVi (mL/m^2^)	27±2	30±2	0.055
LV stroke volume index (mL/m^2^)	35±1	32±1	0.083
Myocardial strain (mid-level:Eu)	−18±1	−16±1	0.003

Values expressed mean ± standard error.

Abbreviations: LV, left ventricular; LVEDV, left ventricular end diastolic volume, LVEDVi, left ventricular end diastolic volume index; LVESV, left ventricular end systolic volume; LVESVi, left ventricular end systolic volume index.

## Summary

This study evaluates whether statin therapy for primary or secondary prevention of cardiovascular disease (CVD) ameliorates declines in left ventricular ejection fraction (LVEF) observed during receipt of anthracycline-based chemotherapy (Anth-bC). We performed magnetic resonance measurements of LVEF before and 6 months after initiation of Anth-bC for patients with breast cancer, leukemia, or lymphoma after accounting for age and sex. Individuals receiving statin therapy experienced less deterioration in LVEF relative to those not receiving a statin upon receipt of Anth-bC.
